# Cochlear implant cost analysis in adults: a European narrative review

**DOI:** 10.1007/s00405-024-08591-3

**Published:** 2024-03-23

**Authors:** Annalisa Gatto, Margherita Tofanelli, Giorgio Valentinuz, Anna Mascherin, Ludovica Costariol, Serena Rizzo, Daniele Borsetto, Paolo Boscolo-Rizzo, Giancarlo Tirelli

**Affiliations:** 1 Department of Medical, Surgical and Health Sciences, Section of Otolaryngology, University of Trieste, Strada di Fiume 447, 34129 Trieste, Italy; 2 Department of Economic, Business, Mathematical and Statistical Sciences ‘Bruno de ‘Finetti’, University of Trieste, Trieste, Italy; 3 Department of ENT, Addenbrookes Hospital, Cambridge University Hospitals NHS Foundation Trust, Cambridge, CB2 0QQ UK

**Keywords:** Cochlear implant, Cost-effectiveness, QALY, Single-sided deafness, Hearing loss

## Abstract

**Purpose:**

The aim of this study was to provide an updated European narrative review spanning the last decade, focusing on the cost-effectiveness of cochlear implants (CIs) for adults with severe to profound post-lingual hearing loss.

**Methods:**

This review encompasses both prospective and retrospective approaches, as well as cross-sectional and longitudinal trials conducted on CIs in adults. All studies related to European countries (Austria, Germany, Switzerland, the Netherlands, Sweden, the UK and Poland) were conducted in English and were published between 2012 and June 2023.

**Results:**

Nine studies were included in the analysis. The patients’ ages ranged from 18 years to over 67 years, with sample sizes ranging from 20 to 100 patients; two of these studies were focused on single-sided deafness in adults. The Markov model was identified as the most commonly utilized analysis method.

**Conclusions:**

This review identified a general consensus on CI cost-effectiveness, despite substantial variability among countries in factors such as observation time horizons, cost-effectiveness thresholds, methods of cost collection, discount rates, CI eligibility criteria and country-specific health systems. Generally, CIs yield positive societal benefits for working-age individuals, potentially less for seniors. Early unilateral CI enhances cost-effectiveness, highlighting the importance of prompt candidate identification. A consistent undersupply of CIs relative to the percentage of potential recipients emerged across countries. Therefore, further investigation into subcategories such as single-sided deafness is warranted, along with country-specific cost analyses. Emphasizing the significance of detailed information on health systems and associated costs and benefits is crucial for facilitating comparisons across different settings.

## Introduction

Nearly 1.5 billion people have hearing loss worldwide, and this incidence is expected to increase in developed countries due to population aging [1]. Unaddressed hearing loss has far-reaching consequences, adversely impacting quality of life, language development, social well-being, educational attainment, and occupational opportunities [2, 3].

Cochlear implants (CIs) represent a well-established means of treating severe-to-profound hearing loss in patients who do not benefit from traditional hearing aids; however, unlike hearing aids, CI necessitates a surgical procedure and incurs some costs throughout the lifetime of the recipient [4, 5]. Over the last 20 years, both CI producers have constantly developed their innovations, and the candidacy has expanded based on more sophisticated methods of diagnosis and increased levels of benefit observed with implantation [6]. CI causes valuable quality of life (QoL) improvement, particularly in terms of speech recognition and reducing levels of isolation, cognitive decline and depression one year post-CI, without mutual correlation [7]. Nevertheless, the direct and indirect costs related to this intervention remain important burdens that should be considered when making policy decisions. One of the first economic analyses investigating CI cost–utility resulted in the inclusion of CI in the funding schedule of California in 1999 [8].

Over the years, in adults, unilateral CI has been shown to be both clinically effective and cost effective [9], while bilateral implantation is still under evaluation regarding costs; nevertheless, expanding guidelines for CI candidacy requires continuous upgrading. The economic evaluation of cost and benefit is a complex process that should consider the differences in the threshold at which an intervention is considered cost-effective among different countries; this threshold is defined as the payer’s willingness to pay. The cost analysis and the consequent threshold may depend on how the health system (and/or the specific interventions) is financed: in the case of public healthcare, the expenditure can be significantly different from that of a private system based on insurance payments. In some countries (Austria, Germany, Poland, Sweden and the UK), the cost is covered by the public system (social insurance, national health insurance) [1, 10–12]; in Switzerland, most of the cost is covered by mandatory health insurance and social security, but a small co-payment is required for patients [13]; whereas in the Netherlands, insurance companies negotiate with all hospitals on the price they are willing to pay for full treatment of a certain disease or diagnosis [14, 15].

In 2011, Nadège et al. conducted a review to analyse the different methods used in cost-analysis CI studies, and they highlighted the poor consensus of methodological approaches, encouraging further cost studies to support the political process as well as the management functions at different levels of healthcare organizations. In fact, cost analysis should be able to identify the actual clinical management of illness and to measure true costs [5].

A recent exhaustive narrative review edited in 2017 analysed the state of the art in terms of both health-related QoL and cost-effectiveness in CI patients [16]. The authors found that considerable work has been done on the QoL status and that the calculated cost–utility ratios have consistently met the threshold of cost acceptance, indicating adequate values for expenditures on CI. Nevertheless, in this regard, the authors did not find European studies on savings and cost utility that were conducted on adults after 2009. These results were confirmed by Turchetti et al. in a previous systematic review conducted in 2011 in which only four articles were selected. The authors highlighted a lack of uniformity among countries and samples, study design, follow-up, utility measures, and cost components; thus, it was difficult to compare the results to perform a quantitative synthesis of the results through meta-analysis [17].

As economic evaluation in individuals with CIs is currently a growing issue, the aim of the present study is, therefore, to provide a European narrative update over the last decade in terms of the cost-effectiveness of CI for adult recipients with severe to profound post-lingual hearing loss.

## Materials and methods

In this narrative review, we incorporated both prospective and retrospective study designs, as well as cross-sectional and longitudinal trials, all of which involved European countries; these studies were carried out in the English language and were published from 2012 to June 2023.

### Participants

Studies that enrolled participants with either pre-lingual or post-lingual onset of hearing loss, including adults (18–65 years) and older adults (> 65 years), were selected; in articles regarding both children and adults, data pertaining to the adult population were considered separately from the data about children and were excluded. No exclusions of the aetiology of hearing loss, duration of hearing loss, timing of CI, or duration of follow-up were made.

### Outcome measures

The following primary outcomes were reported in the identified studies:

1. Costs and savings associated with mono- or bilateral CI;
2. Cost-effectiveness, cost‒benefit, or cost–utility of CI

### Search methods

To include all the actual literature on CI studies, the main English language electronic biomedical publication databases were screened (PubMed, EMBASE, CINAHL, Web of Science and economic periodicals). The words we used for the research included variations in the terms “cochlear implantation” and “cost-effectiveness”: “cochlear implant” AND “cost study” OR “cost analysis” OR “cost evaluation” OR “economic evaluation” OR “economic analysis”; we applied restrictions on the articles for country of origin, including only European studies. All studies published in English that assessed the costs of CI were selected.

Studies were excluded if they were editorials, case reports, or non-English language publications; if the reviews were excluded from the study, their references were accurately screened to ensure that valuable and citable works were not overlooked.

## Results

**Fig. 1:**
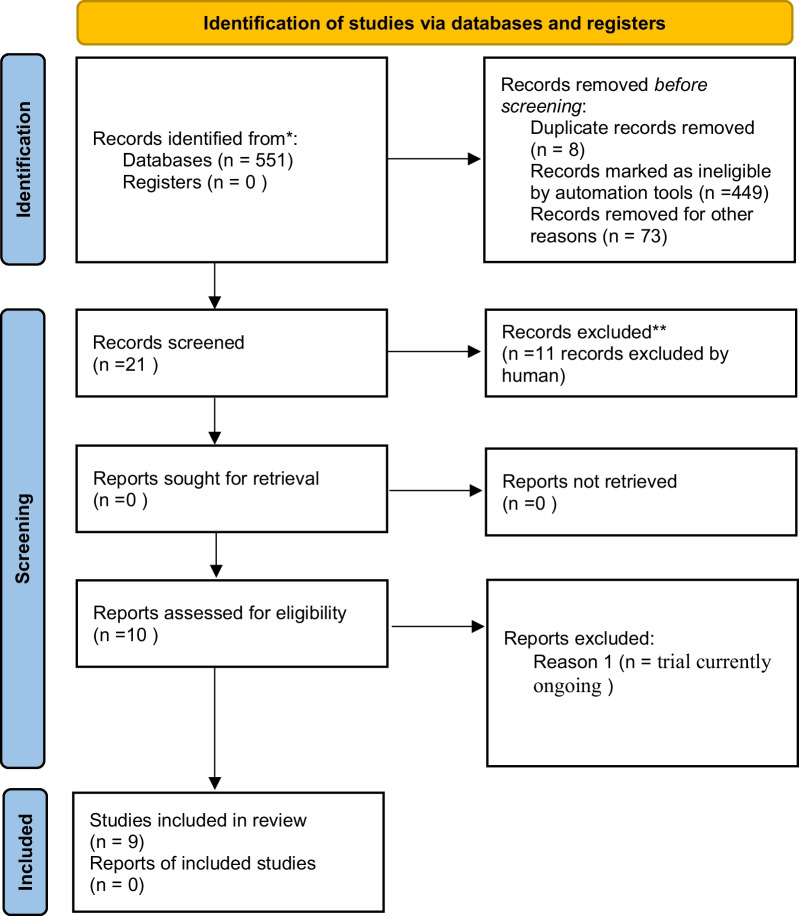
PRISMA 2020 flow diagram which included searches of databases and registers only

Our search yielded 551 studies conducted over the last 10 years, 531 of which were in English. Among these, 106 pertained to European countries. Focusing on these, we identified 52 studies that included adult populations. We excluded 8 duplicate studies and 23 studies that did not address the costs of cochlear implantation. Initially, 21 studies were selected for closer examination. After a thorough full-paper review of these 21 studies, we ultimately included 10 papers in our analysis. However, one of these papers was subsequently excluded [18] because the trial (phase II) is currently ongoing; thus, 9 studies were included. The PRISMA diagram visually represents the flow of the literature review process (Fig. 1). Table 1 reports the main features of each paper that has been included (see Table 1).

**Table 1:** Summary of the main features of each included study

Study	Sample size (n), gender (n female), mean age at CI/time of study	Geographic origin	Type of study	Model objective	Primary benefit	Cost analysis	Results
The cost-effectiveness of cochlear implants in Swedish adults Gumbie et al. (2021) [1]	38 adults aged 19 years and older, with an average age of 61 years, who had previously received benefits from a hearing aid	Sweden	CIs cost effectiveness vs hearing aids	To estimate: ICER (Using a cost-effectiveness threshold of SEK 250,000 per QUALY gained) in different scenarios	HUI3 Unilateral CI vs no intervention: utility increment of 0.210 (literature derived); increased health-related QoL by 3.10 QALYs on average	ICER (per QALY) in Unilateral CI vs HA Base case: 140,474 SEK Lowering the age at 50 years: 118,232 SEK Frequency of sound processor upgrade every 5 years: 163,169 SEK Proportion eligible after triage at 70%: 136,964 SEK Multiway scenarios: 138,751 SEK	Unilateral CI is a cost-effective option. Earlier implantation improves the cost-effectiveness, as does improved identification of people eligible for CIs
Direct cost of cochlear implants in Germany—a strategic simulation Thum et al. (2022) [20]	Adult population (20 years or above) for 40 years (2018–2057)	Germany		To estimate future CI demand and costs	Feasibility: unilateral from 76.57 (20–29 y) to 27.30% (90 + y); Bilateral CI from 82.81% (20–29 y) to 30.94 (90 + y) Willingness: unilateral CI from 68.64% (20–29 y) to 13.12 (90 + y); bilateral CI from 72.92% to 10.32% (90 + y)	According to the simulation, it’s expected a total cost of about 21.5 billion € for CI care in the forecast period (2018–2057) Increasing willingness scenario: 222 billion EUR (2018–2057), + 887.2%	CI demand and cost supply (+ 40%) will increase in the future
The cost-effectiveness of unilateral cochlear implants in UK adults Cutler et al. (2021) [11]	UK adult population with severe to profound hearing loss who had previously received benefits from a hearing aid and adults who had not	UK	UCI versus a hearing aid and a UCI versus no hearing aid	To estimate: differences in lifetime benefits and costs associated with a UCI	QALY: Unilateral CI vs. HA: gain 3.18 Unilateral CI vs no HA: gain 3,66	Average incremental lifetime cost: Unilateral CI vs. HA: £37,988 Unilateral CI vs. non HA: £38,449 ICER (per QALY): Unilateral CI vs HA: £11,946 Unilateral CU vs no HA: £10,499	Unilateral CI remain cost-effective despite changes to clinical practice and increased healthcare unit costs. The future increased access to CI will improve QoL of recipients and overall social welfare
Cost–benefit Analysis of Cochlear Implants: A Societal Perspective Neve et al. (2021) [14]	Meta-analysis of Netherland's majority of cochlear implant patients: prelingually deaf children implanted at the age of 1 year; group 2: working age adults (age 18–67), implanted at the age of 40; Group 3: retired seniors (> 67) implanted at the age of 70	Netherlands	Bilateral CI simultaneous vs no treatment; bilateral CI simultaneous vs unilateral CI	To estimate: ICER (threshold of 50 000 EUR) and the net benefit	HRQL using HUI3: Adults and senior: gain of 0,17 for unilateral CI vs no intervention; gain of 0,21 for bilateral CI vs no intervention Incremental QALY:	ICER (per QALY): unilateral CI vs. no intervention: Group 2: 200 EUR; net benefit 275,000 EUR; Group 3: 23,000 EUR, net benefit 76 000 EUR unilateral CI vs. bilateral CI: Group 2: 64,000 EUR, net benefit—16,000 EUR Group 3: 220,000 EUR, net benefit—45,000 EUR Bilateral vs. unilateral CI not cost-effective	The indication of a CI at any age during a person's working life and at senior age had a positive net benefit from the societal perspective; early treatment led to an higher societal benefits
Cost–utility Analysis of Cochlear implant in Adults With single-side Deafness: Austrian and German Perspective Seebacher et al. (2021) [10]	20 patients with SSD (> 80 dB HL) aged 18 years or older, implanted for the first time (literature derived)	Austria and Germany	Unilateral CI vs. No treatment	To calculate the ICUR	QoL measure: HRQoL data derived from HUI-3 (literature derived)	Unilateral CI vs. no intervention ICUR (per QUALY): Austrian patients cohort: 34,845.2 EUR German patients cohort: 31,601,25 EUR	CI is a cost-effective treatment option for adults with SSD The duration of CI use had a significant effect on the incurred costs, whereas costs get lower for longer periods of CI use
Cost–Utility Analysis of Bilateral Cochlear Implantation in Adults With Severe to Profound Sensorineural Hearing Loss in Poland Skarzynski et al. (2021) [12]	22 adult patients (59.1% men, 40.9% women) aged from 59.13 ± 8.9 years	Poland	Bilateral CI simultaneous vs bilateral CI sequential 'short delay' vs bilateral CI sequential 'long delay' vs no treatment	To calculate ICUR	QoL measure: AQoL-8D questionnaire DeltaQALY: 1.21 in every scenario	ICUR (per QALY): Short delay: 236 804.09 PLN Long delay: 232 564.94 PLN No delay: 227 414.8 PLN	Bilateral CI strategies are cost-effective compared to no intervention. When the 3 model scenarios are compared the bilateral simultaneous CI is even more cost-effective
Age Dependent Cost-Effectiveness of Cochlear Implantation in Adults. Is there any Age related Cutoff? Laske RD et al. (2019) [13]	*n* = 100 adults (mean age 51)	Switzerland	Unilateral CI vs bilateral hearing aids; sequential bilateral CI vs bilateral hearing aids; and sequential bilateral CI vs unilateral CI	To calculate the ICER and how it vary depending on patient’s age	Unilateral CI vs HA: gain 0.28 Sequential CI vs. HA: gain 0.38 Sequential CI vs. Unilateral CI: gain 0.1	Unilateral CI vs. HA: cost-effective up to an age of 91 for women and 89 for men Sequential CI vs HA: cost-effective up to an age of 87 for women and 85 for men Sequential CI vs Unilateral CI: cost-effective up to an age of 80 for women and 78 for men	Performing both sequential and unilateral CI is highly cost-effective up to very advanced ages when compared with HA The younger a patient is at implantation, the longer he or she will enjoy an improved QoL
Cost–utility of bilateral versus unilateral cochlear implantation in adults: a randomized controlled trial Smulders et al. (2015) [15]	38 postlingually deafened adults (18–70 years)	Netherlands	Unilateral versus simultaneous bilateral CI	To calculate ICUR	Utility measure: HUI3 TTO VAS on hearing VAS on general health EQ-5D	Direct cost: Unilateral CI: EUR 43,883 + –EUR 11,513(SD) Bilateral CI: EUR 87,765 + –EUR 23,027(SD) Annual costs: Unilateral CI: EUR 3435 + –EUR 1085(SD) Bilateral CI: EUR 6871 + –EUR 2169(SD)	A second implant became cost-effective after 5–10 year period
Cost Effectiveness of Cochlear Implantation in Single-sided Deafness Dreyfuss et al. (2021) [19]	Adults with acquired single-sided deafness and tinnitus	Switzerland	Unilateral CI vs. No treatment	To estimate the ICER	Utility measure: literature derived HUI3, SSQ (Arndt et al. and a multiple linear regression model to predict HUI3 values based on SSQ) Mean HUI3: No intervention 0.62 vs Unilateral CI 0.74	The ICER using the Arndt estimate at young patient ages was approximately half of the ICER using the regression estimate	CI is a cost-effective option for patients with SSD: women up to 64, 80 m and 86 years; men up to 58, 77 and 84 The younger a patient was at implantation, the intervention more cost effective

Globally, the patients’ ages varied from 18 years to > 67 years, and the sample size ranged from 20 to 100 patients.

These studies were conducted in northern Europe (Austria, Germany, Switzerland, the Netherlands, Sweden, the UK and Poland); two studies have focused on single-sided deafness (SSD) in adults [10, 19], one study has been developed as a simulation to forecast costs for 40 years from a social health insurance perspective [20] and the other six have estimated bilateral and/or unilateral CI cost-effectiveness in adults [1, 11–15].

Only one study included a group of children, which was separately analysed in the text and not included in this review [14].

The Markov model has been the most commonly used analysis method.

### Unilateral CI in adults

The cost analysis indicated that CI is more cost-effective than hearing aids from both societal and patient perspectives. Specifically, recommending a CI at any age during a person’s working life has a positive net benefit from the societal perspective, while the cost-effectiveness of CI for seniors is somewhat lower [1, 11, 14]. Considering age variations in the Swiss context, performing unilateral CI is cost-effective up to the age of 90, while sequential CI remains cost-effective up to ages 87–85. Using a threshold of 100,000 CHF per quality-adjusted life year (QALY), sequential CI, compared to unilateral CI, is cost-effective up to ages 78–80 [13].

Additionally, for SSD in adults in Germany, the cost-effectiveness of CI was observed in comparison to no treatment [10, 19].

### Bilateral CI in adults

Simultaneous bilateral CI becomes a cost-effective intervention after 10 years. Notably, this approach proves to be cost-effective when performed both simultaneously and sequentially compared to no intervention. No significant differences emerged when different surgical timings were used [12, 15]. Notably, in the UK [11] and the Netherlands [15] bilateral cochlear implantation is not reimbursed for adult patients (over 19 years old); similarly, in Poland, simultaneous cochlear implantation is not covered by social security [12].

General costs can be classified into direct measurable costs and other benefits derived from IC implantation.

Direct costs were assessed to evaluate the following:

- Unilateral CI compared to hearing aid or no hearing aid
- Unilateral vs bilateral CI costs or vs second sequential CI
- Comparison between bilateral simultaneous strategy and bilateral sequential CI costs
- Annual costs from the second year onwards
- Simulation of future costs in a forecasted period of time

The most common approaches used in studies evaluating the goodness of fit (even from different perspectives) of treatments are as follows:

- Incremental cost-effectiveness ratio (ICER) or incremental cost–utility ratio (ICUR): defined as the difference in costs (ore benefits) between two possible interventions divided by the difference in quality-adjusted life years (QALYs);
- Cost effectiveness acceptability curve (CEAC): the probability that an intervention is cost-effective when compared with the alternative for a specific or a range of values of cost-effectiveness thresholds;

Tables 2, 3 report the comparisons among the enrolled studies in terms of economic evaluation.

**Table 2:** Economic data and cost utility results from included studies - part I

#	Study	Geographic origin	Type of healthcare system	Time covered	Valuta	Perspective	Design of the analysis	Discount rate	Follow up (years)	QALY cost-effectiveness threshold	Access to Cis decision	Sources of unit costs
1	Gumbie et al. (2021) [1]	Sweden	CI are publicly funded in Sweden to patients with severe or profound hearing loss	n/a	2019 Swedish Krona prices (SEK 1 = EUR 0.09521 on 31/12/2019)	Swedish healthcare payer	Markov model with a 6-month cycle length, considering disutility too, short term and long term	3%	Lifetime horizon to capture all differences in lifetime costs and health outcomes following unilateral CI surgery	SEK 250.000 per QALY gained (approximately EUR 23,800)	Candidacy criteria set by the Swedish government	Swedish National Board of Health and Welfare, Swedish Association of Local Authorities and Regions and clinical expert opinion
2	Thum et al. (2022)[20]	Germany	The article was not included, as it presents an evaluation framework for the entire country at a macro and aggregate level									
3	Cutler et al. (2021) [11]	UK	CI are funded by the National Health Service (NHS)	n/a	2018 UK pound prices (GBP 1 = EUR 1.1139 on 31/12/2018)	UK NHS	Markov model with a 6-month cycle length, considering adverse events and failures too, short term and long term	3,5%	Lifetime horizon to capture all differences in lifetime benefits and costs associated with a UCI	GBP 20,000 per QALY gained (approximately EUR 22.278)	Eligibility criteria according to NICE	Clinical expert opinion, literature reviews, NHS National Schedule of Reference Costs, NHS National Tariff: Currencies and Prices, the Personal Social Services Research Unit, and literature
4	Neve et al. (2021) [14]	Netherlands	The charge is negotiated between hospitals and health insurance companies		EUR	Costs and benefits analysis of CI from a broader societal perspective:health outcomes, healthcare cost, educational cost, and productivity losses and gains	Markov state transition model with model parameters and assumptions based on published literature. Probabilistic and one-way sensitivity analyses were performed	Future costs and health outcomes were discounted at annual rates of 4% and 1.5%, respectively	Expected lifetimes of the members of each group	According to Dutch reference values, a QALY is valued at EUR 20,000, EUR 50,000, or EUR 80,000, depending on a low, medium, or high burden of disease		Published literature was used for the input of the CBA. If unavailable; the input was based on the opinions of a panel of experts. No human subjects were involved. Average charges of all hospitals in 2018 published by the Dutch Health Authority were used as a proxy for the real healthcare cost
5	Seebacher et al. (2021) [10]	Austria and Germany	CI are covered by the National health insurance	2 years (2018–2019)	2019 EUR	A third-party payer perspective was adopted: only costs related to the intervention and those covered by the national health insurance were taken into account	Markov model that compares two alternatives (unilateral CI vs no treatment). Only for Austria data set was available in full detail	3%	20 years	No explicitly stated willingness-to-pay (WTP) thresholds for Austria		Hospital data and sickness fund data
6	Skarzynski et al. (2021) [12]	Poland	CI are covered by the Polish Social Insurance Institution (except for the simultaneous cochlear implantation)		2019 Poland Zloty (1 PLN = 0.2352 EUR on 31/12/2019)	A third-party payer perspective was adopted: only costs covered by the Polish Social Insurance Institution were considered	Markov model to compare no treatment vs bilateral CI. Three different timings for CI were used (simultaneous implantation is theoretical)	3.5% for utility values; 5% for cost data	10 years			Diagnosis Related Group data; data from clinical trial
7	Laske RD et al. (2019) [13]	Switzerland	Mandatory health insurance and social security covers most of the costs. A small co-pay is paid by the patient	2010–2013	2017 Swiss Francs (1 CHF = 0.85508 EUR on 31/12/2017)		Cost–utility analysis using a Markov model: Unilateral CI vs no intervention (HA), Sequential CI vs no intervention (HA), Sequential CI vs. Unilateral CI	0%. Different discount rates (1, 2, 3 and 5%) are used in the simulation	All the costs and health utility values were calculated over the expected patient’s lifetime	CHF 50,000 (EUR 42,754) and CHF 100,000 (EUR 85,508) from literature		Cost parameters were collected for the year 2017 in a public tertiary referral hospital setting in Switzerland
8	Smulders et al. (2015) [15]	Netherlands	In the Netherlands, insurance companies negotiate with all hospitals on the price they are willing to pay for a treatment. Bilateral implantation is reimbursed for children (up until 19 years), but not for adult patients	01/2020–09/2012	2013 EUR	Dutch health insurance		0%	After 1 and 2 years. analysis of QALYs were conducted for different periods, with a maximum of 25 years	Willingness-to-pay is between EUR 24,500 and EUR 80,000 per QALY, dependent on the seriousness of the disease		Five University Medical Centers (Utrecht, Maastricht, Leiden, Nijmegen and Groningen) with a different number of patients each. The authors used proxies given by the amount’s noninsured patients are charged for CI and published on each hospital’s website
9	Dreyfuss et al. (2021) [19]	Switzerland	Mandatory health insurance and social security covers most of the costs. A small co-pay is paid by the patient (information from another article)		2019 CHF transformed in USD at the exchange rate of 1 USD = 1 CHF (1 CHF = 0,9198 EUR)		Cost–utility analysis using a Markov model	Different discount rates are used in the simulation: 0, 1, 3, and 5%		ICER threshold of USD 50,000/QALY—from the literature—was used (EUR 45,992/QALY)		Cost parameters of a public tertiary referral hospital setting in Switzerland

*Considering the 'absolute' cost rather than the incremental cost compared to traditional support, in order to enable comparisons across different studies

*Probabilistic Sensitivity Analysis

**Table 3:** Economic data and cost utility results from included studies - part II

#	Study	Geographic origin	Valuation	Sensitivity	Scenarios/Variables	Total cost	QALY	ICER	Device cost
1	Gumbie et al. (2021) [1]	Sweden	CI are publicly funded in Sweden to patients with severe or profound hearing loss	n/a	2019 Swedish Krona prices (SEK 1 = EUR 0.09521 on 31/12/2019)	Swedish healthcare payer	Markov model with a 6-month cycle length, considering disutility too, short term and long term	3%	Lifetime horizon to capture all differences in lifetime costs and health outcomes following unilateral CI surgery
**2**	Thum et al. (2022)[20]	Germany	The article was not included, as it presents an evaluation framework for the entire country at a macro and aggregate level						
3	Cutler et al. (2021) [11]	UK	CI are funded by the National Health Service (NHS)	n/a	2018 UK pound prices (GBP 1 = EUR 1.1139 on 31/12/2018)	UK NHS	Markov model with a 6-month cycle length, considering adverse events and failures too, short term and long term	3,5%	Lifetime horizon to capture all differences in lifetime benefits and costs associated with a UCI
4	Neve et al. (2021) [14]	Netherlands	The charge is negotiated between hospitals and health insurance companies		EUR	Costs and benefits analysis of CI from a broader societal perspective:health outcomes, healthcare cost, educational cost, and productivity losses and gains	Markov state transition model with model parameters and assumptions based on published literature. Probabilistic and one-way sensitivity analyses were performed	Future costs and health outcomes were discounted at annual rates of 4% and 1.5%, respectively	Expected lifetimes of the members of each group
5	Seebacher et al. (2021) [10]	Austria and Germany	CI are covered by the National health insurance	2 years (2018–2019)	2019 EUR	A third-party payer perspective was adopted: only costs related to the intervention and those covered by the national health insurance were taken into account	Markov model that compares two alternatives (unilateral CI vs no treatment). Only for Austria data set was available in full detail	3%	20 years (because different publications report that the performance of the CI is stable for 20 years and more)
6	Skarzynski et al. (2021) [12]	Poland	CI are covered by the Polish Social Insurance Institution (except for the simultaneous cochlear implantation)		2019 Poland Zloty (1 PLN = 0.2352 EUR on 31/12/2019)	A third-party payer perspective was adopted: only costs covered by the Polish Social Insurance Institution were considered	Markov model to compare no treatment vs bilateral CI. Three different timings for CI were used (simultaneous implantation is theoretical)	3,5% for utility values; 5% for cost data	10 years
7	Laske RD et al. (2019) [13]	Switzerland	Mandatory health insurance and social security covers most of the costs. A small co-pay is paid by the patient	2010–2013	2017 Swiss Francs (1 CHF = 0.85508 EUR on 31/12/2017)		Cost–utility analysis using a Markov model: Unilateral CI vs no intervention (HA), Sequential CI vs no intervention (HA), Sequential CI vs. Unilateral CI	0%. Different discount rates (1, 2, 3 and 5%) are used in the simulation	All the costs and health utility values were calculated over the expected patient’s lifetime
8	Smulders et al. (2015) [15]	Netherlands	In the Netherlands, insurance companies negotiate with all hospitals on the price they are willing to pay for a full treatment of a certain disease or diagnosis. Bilateral implantation is reimbursed for children (up until 19 years), but not for adult patients	01/2020–09/2012	2013 EUR	Dutch health insurance		0%	After 1 and 2 years. analysis of QALYs were conducted for different periods, with a maximum of 25 years
9	Dreyfuss et al. (2021) [19]	Switzerland	Mandatory health insurance and social security covers most of the costs. A small co-pay is paid by the patient (information from another article)		2019 CHF transformed in USD at the exchange rate of 1 USD = 1 CHF (1 CHF = 0.9198 EUR)		Cost–utility analysis using a Markov model	Different discount rates are used in the simulation: 0, 1, 3, and 5%	

In the literature, the standard acceptability threshold for deeming an intervention effective is generally set at approximately 25,000 (dollars or Euros) per QALY gained. However, more recent studies have raised this threshold to as high as EUR 100,000 per QALY [13]; in the reviewed articles, the range varies, in fact, from approximately EUR 25,000 to EUR 100,000 per QALY, with a few instances closer to EUR 20,000, potentially influenced by currency exchange rates [1, 11]. Relative to Dutch reference values, a QALY is valued at EUR 20,000, EUR 50,000, or EUR 80,000, depending on the burden of disease—categorized as low, medium, or high [14].

When considering the sources of information used for cost collection, only a few studies utilized data directly obtained from hospitals [10, 13, 19] or websites [15] as a primary source. In contrast, other studies have relied primarily on the literature, documents issued by national health authorities [1, 11], expert panels, or a combination of sources.

The analysed studies employed varying time horizons for the calculation of costs and benefits. Some studies utilized a relatively short time frame, such as 10 years [12] or 20 years [10]. One study employed different time horizons, with a maximum of 25 years [15], while the majority utilized the entire expected life span of the patients [1, 11, 13, 14].

Considering broad time horizons would require the use of discount rates to make future values comparable at the same point in time. The analysed studies made different choices regarding the discount rate. Some used a rate between 3% and 3.5% [1, 10, 11], one chose not to discount future flows at all [15], some attempted to use alternative discount rates in the simulation [13, 19], and others applied different discount rates for costs than for utility values [12, 14]. These varied choices of time and rate make it challenging, on their own and without considering other elements of heterogeneity, to obtain useful and insightful data for comparison among the different countries analysed in the studies.

## Discussion

This review provides an update regarding the cost-effectiveness of CI surgery for adults in Europe. Recently, several countries have conducted internal audits to determine the cost-effectiveness of CI surgery, which is a relatively expensive intervention that is usually performed at tertiary referral institutes [19].

From the results, considerable variability among countries and each healthcare system emerged, thus making comparisons quite difficult. Generally, we observed that most of the cost-effective analyses have been conducted in Northern Europe, focusing on unilateral CI and applying a Markov model to estimate costs and benefits.

Grouping studies based on methodology and comparisons between unilateral CI costs in the adult population and those associated with yes/no hearing aids were performed with all the Markov models [1, 11]. All the authors conducted a cost analysis in which ICERs were estimated and the results agree on the positive cost-effectiveness of unilateral CI finding a trend of incremental gain in terms of QALY and Health Utility Index-3 (HUI-3). While the Markov model is utilized in nearly all studies, the differing degrees of completeness and complexity in assumptions, the exploration of alternative scenarios, the utilization of discrete or probabilistic values, and varying time horizons create challenges for statistically comparing analyses across studies, posing unresolved questions. There is no doubt that the method of calculating costs or benefits, along with the chosen time horizon, can significantly influence the final result.

Neve et al. [14] were the only authors who considered expenditures from a societal perspective outside the healthcare sector, introducing lifetime societal costs. The study concluded that unilateral CI is more expensive than hearing aid but has a 92% likelihood of being cost effective; it is important to emphasize that the value of the likelihood percentage varies with the varying cost-effectiveness threshold fixed in the analysis. The primary challenge in making monetary comparisons among countries stems from this factor. A crucial consideration is that variations in eligibility criteria for CI contribute to disparities in outcomes. Consequently, differences in ICERs may not only reflect economic contrasts but also divergences in study methodologies.

Moreover, many candidates for CI have not yet gotten one, probably because the treatment is elective and requires a patient to receive the implant; other implants, conversely, are usually recommended by specialists, such as peacemakers, at the time of an emergency. Other types of implantable medical devices, such as knee and hip devices, are also elective, but candidacy to CI is regulated differently among countries. In Sweden, the criteria for eligibility are set by the government; in the United Kingdom and Dutch-speaking areas, strict recommendations are followed in the NICE guidelines [1].

Cutler et al. [11] reported that unilateral CI is more cost effective in adults than both hearing aid and no treatment, with 93% and 99% likelihood, respectively, assuming a cost-effectiveness threshold of 20,000£ per QALY. A limitation is that in Sweden, there is no explicit cost-effectiveness threshold for deciding whether to fund an intervention; nevertheless, the authors found that earlier implantation of unilateral CI improves cost-effectiveness, suggesting that the identification of eligible people should also be mindful [1].

Some authors considered all the cost-effectiveness of bilateral CIs; in particular, the majority of the authors compared CI utility versus no intervention or hearing aid [12, 13], while others considered the differences between bilateral simultaneous CIs and unilateral CI [15]. These studies convey that bilateral CIs are cost-effectiveness: Smulders et al. highlighted that simultaneous CI use could be convenient for people with a life expectancy equal to or greater than 5–10 years [15], while Laske et al. found that both sequential and unilateral CIs are cost effective up to very advanced ages when compared with hearing aids, indicating that background cost and age are not contraindications for CI in otherwise suitable patients [13]. We should also take into account that the cost utility of bilateral versus unilateral CI is strongly conditioned by the cost of the second implant, as in some health care settings, it has been heavily discounted, up to 50%, especially in simultaneous surgery, although effectiveness is not always measurable in terms of functional outcomes assessed through questionnaires [15].

Among the included studies, Thum et al. utilized a system dynamics model to project the volumes and direct costs of cochlear implant interventions in adults over a 40-year period, extending through 2057. This analysis was conducted from the perspective of social health insurance [20]. This method is a standard mathematical prognostic approach that the authors used to simulate an increase in CI demand and cost for different scenarios: at baseline, in a higher willingness for CI, in a more extended indications for CI and in a scenario of CI technological innovation. The mathematical models estimated that the annual CI supply cost would increase by 16% over the next 40 years; thus, gradual process optimization may lead to a cost-saving effect.

Finally, considering the cost–utility of CI in single-sided deafness, only direct costs according to the Swiss health-care system and from Austrian and German perspectives were analysed [10, 19]. In the German population, the cost analysis assumed different cost-effectiveness thresholds stressing the effect of age at the time of intervention; thus, the ICERs vary for each age/sex combination. The authors concluded that, relative to their background and by adopting a threshold of 100,000 dollars, CI implants are cost effective for elderly individuals (> 70 years old) based on a life expectancy at birth of 80 years for men and 84 years for women; additionally, the analysis has been conducted by extrapolating data from the literature and utility indices based on the average study population, and no comparison with an alternative treatment has been performed [19]. In contrast, Seebacher et al. assessed the QoL in a cohort of 20 Austrian and German patients affected by SSD, and the model was used to compare the group receiving a CI versus the group receiving no intervention, confirming that cochlear implantation is a cost-effective option for patients with SSD within the Austrian and German health care systems [10].

## Conclusions

The present review highlights that a general cost-effectiveness of CI emerged from all the studies, but a comparison among countries and different types of intervention remains challenging due to the large amount of heterogeneity both in health systems and country-specific ICUR thresholds that derive from the region-specific gross domestica product pro capita (GDPPC), as well as the way in which the system is financed by the type of intervention and the availability of information to support the studies, which may vary both from country to country (e.g., in the Netherlands, much of the cost information is confidential [16]) and the way it is collected (direct or literature analysis). Moreover, there is a need to investigate in depth the subcategory of SSD that was identified by the update published in 2019 by the National Institute for Health and Care Excellence (NICE) [6]. A similarity has been observed across different countries: there is a consistent undersupply of cochlear implants (CIs) relative to the percentage of potential recipients. Only slightly less than 10% of individuals meeting the criteria for cochlear implants (CIs) have access to this option, despite CI implantation being a well-established standard procedure for severe to profound hearing loss [21, 22].

We therefore encourage further country-specific cost analyses, emphasizing the importance of detailed information on the health system and all associated costs (and benefits) to facilitate meaningful comparisons among different settings. This approach will facilitate meaningful comparisons across different backgrounds, considering also the cost-effectiveness threshold chosen and the patient age as key points. In light of the literature reviewed, we propose a comprehensive list of cost elements to be considered in all analyses. This list aims to standardize the evaluation criteria and ensure a thorough and consistent approach to cost analysis in CI healthcare research.

## KEY-POINTS BOX: Proposed Outcome and Cost Reporting

- Declare the adopted time horizon of the analysis: 10y, 20y, >25 years; if using a lifetime horizon, provide analysis results for 10 or 20 years for comparability with existing literature.
- Apply homogeneous thresholds for deeming an intervention effective: universally accepted at 25,000/50,000 Euros or dollars.
- Provide a detailed description of sources of information used for cost collection, ensuring to specify whether direct or indirect costs have been considered.
- Describe the health system context in which the study is conducted.
- Adopt a fixed discount rate within the range of 3-3.5%.
- Follow uniform eligibility criteria for cost-effectiveness analysis, preferably according to NICE 2019 guidelines.

## Declarations

### Research involving human and/or animal participants

The study did not involve human participants and/or animals.
